# Sustainable Paper-Based Packaging: A Consumer’s Perspective

**DOI:** 10.3390/foods10051035

**Published:** 2021-05-10

**Authors:** Omobolanle O. Oloyede, Stella Lignou

**Affiliations:** Sensory Science Centre, Department of Food and Nutritional Sciences, Harry Nursten Building, University of Reading, Whiteknights, Reading RG6 6DZ, UK; bola.oloyede@reading.ac.uk

**Keywords:** paper-based packaging, consumers, focus groups, sustainability, environmentally friendly

## Abstract

Over the last two decades, there has been growing interest from all stakeholders (government, manufacturers, and consumers) to make packaging more sustainable. Paper is considered one of the most environmentally friendly materials available. A qualitative study investigating consumers’ expectations and opinions of sustainable paper-based packaging materials was conducted where 60 participants took part in focus group sessions organized in two stages. In the first stage, participants expressed their opinions about currently available packages in the market and their expectations about a sustainable packaging material. In the second stage of the study, they evaluated five paper-based prototype packages for two product categories (biscuits and meat). Too much plastic and over-packaging were the key issues raised for current packages. Price and quality were the main driving forces for consumers’ purchase intent. While participants were impressed by the sustainable nature of the prototypes, the design did not necessarily meet their expectations, and they were not willing to pay more for a sustainable package. The key message that emerged from the discussions was the “3Rs”—Reduce, Reuse, and Recycle”—which should be the main points to consider when designing a sustainable packaging.

## 1. Introduction

The role of packaging in the safe delivery and transportation of products across the food chain cannot be overemphasized. To prevent food waste and loss, a good food package should ensure that food quality and safety is maintained from transportation through to storage of the product [1]. However, a major disadvantage of packaging is that it adds to the world’s environmental footprint because it is always discarded immediately after the product is used [2]. The main types of materials used for food packaging include paper (including cardboard), wood, glass, metal, and various types of plastics.

Over the last two decades, there has been growing interests from governments, manufacturers, and consumers to make packaging more sustainable. Recent research in packaging focused on sustainability and how to make packaging materials more eco-friendly [3,4]. Technically, sustainable packaging has been defined as a packaging with a relatively low environmental impact based on life-cycle assessments (LCA) [5]. However to the average consumer, a sustainable package can be considered “a packaging design that evokes explicitly or implicitly the eco-friendliness of the packaging” [6].

Paper as a packaging material is experiencing a revival, as consumers perceive it as a high-value and environmentally friendly material [7,8,9]. Paper has the advantage of being bio-based, biodegradable, and recyclable. Studies from the Institute for Energy and Environmental Research (Germany) showed a significantly lower impact of paper-based packaging on the environment compared to many other materials. Globally, paper-based packaging has the potential to tackle marine debris and lead to a lower impact of packaging in the environment. This is especially necessary as the amount of packaging used is steadily increasing due to small portion packaging, urbanization, and a growing worldwide population.

In food packaging, there are various opportunities for more paper and a reduced polymer content; however, the technology has to be adapted to the production process, and the material composition has to fit to the product requirements. The possibilities of paper packaging are progressing, and solutions for barrier properties and formability are being addressed.

While life-cycle assessments (LCAs) show the sustainability value of packaging, it is important to understand consumer opinion and perception of these packages if marketing is to be successful. This is because consumer opinions and beliefs of a package which influence choice and purchase are not determined by LCA results [7,10]. The success of environmentally friendly packages is largely dependent on consumers as they are the ones who determine whether or not to buy the packages [11]. To increase consumer acceptability and purchase of sustainable packages, a detailed understanding of their opinions and perceptions of environmentally friendly packaging is needed [2].

A recent review by Ketelsen et al. [11] found only 21 out of 46 studies reviewed were focused on consumer responses to environmentally friendly packaging, showing that this area of research is not very well explored and demonstrating the need for more research in this area.

While some studies previously focused on the effect on perceived product quality of sustainable packaging [12,13], others focused on the influence of the design and labelling elements on consumer perceptions on environmentally friendly packaging [6,13]. Ertz et al. [13], in their study investigating the influence of environmental information on the reaction of 321 Canadian consumers, found that consumer perception of product quality was enhanced when environmental claims and labelling cues were well defined on the product packaging. However, when an environmental label was not accompanied by detailed self-declared environmental claims, the perception of product quality was not significantly enhanced.

Consumer awareness of the environmental impact of food packaging has been studied [9,14,15]. Participants who took part in focus group discussions and a survey in Italy considering labelling information on packaging stated that there was currently no information about environmentally friendly characteristics on packages and showed a high interest in having information about the sustainable characteristics of the packaging [15]. In their study on consumer responses to packaging design, Steenis et al. [9] reported that having sustainability cues on packaging was a key factor in determining how packages differed as evaluated by university students in the Netherlands.

A study conducted by Scott and Vigar-Ellis [16] on consumer understanding, perceptions, and behaviors in relation to environmentally friendly packaging in South Africa found that consumers had limited knowledge of what environmentally friendly packaging is, how to differentiate it from other packaging, as well as what benefits different packaging had. South African consumers stated that labels, images, and logos were the most important features used in helping them identify the environmentally friendly packaging. The packaging material and its color were other features used to judge packaging sustainability.

Consumer preference and willingness to buy or purchase products with environmentally friendly packaging was previously relatively well studied with conflicting results [17,18,19,20,21,22]. In a study conducted by Rokka and Uusitalo [17], where they compared green packaging with several product attributes and how these attributes affect consumer environmental choice, they found that one-third of the consumers participating in the study agreed that one of the most important criteria in their choice was the environmentally labelled packaging. Jerzyk [22] explored the attributes of sustainable packaging that have a positive impact on consumer behaviour and how purchasing intentions can be influenced when the packaging is sustainable among Polish and French students. They reported that sustainable packaging is not the most important factor when buying a product and that students are not willing to lose any of the functional and quality characteristics of the products because of the sustainable nature of the packaging. Concern for the environment and beliefs was shown to have an impact on purchase intent of eco-friendly packaging. Previous studies showed that consumers that are generally concerned about the environment are more likely to buy sustainable packaging [23,24,25,26].

In most cases, studies focused on environmentally friendly packaging in general rather than on specific packaging solutions [6,7,16,22,26,27,28]. Very few studies, however, focused on specific packaging for specific products such as paper packaging for cereal bars [13], glass packaging for foods [21] and for milk [29], and various packaging materials for tomato soup products [9]. This shows that existing knowledge on consumer responses to specific sustainable packaging solutions is limited. Thus, Ketelsen et al. [11] recommended that future research should focus on specific packaging solutions rather than environmentally friendly packaging in general to provide a deeper understanding of consumer opinions and acceptability of specific solutions. Focus groups, surveys, and interviews are some of the methodologies that were previously used to explore consumer insights in research. Focus groups which are generally used at the earlier stages of consumer research were used by several authors as they have the main advantage of allowing freedom of expression and open discussions from participants [30,31,32]. In light of this, the objectives of this study were to: (i) understand consumer perception of currently available food packaging; (ii) design sustainable paper-based packages for biscuit and meat products based on consumer opinions and expectations of sustainable paper-based packaging over a series of participatory focus group sessions; (iii) understand consumer opinions of the paper-based packages developed as well as evaluate and assess the characteristics and suitability of the packages. The rest of paper is divided into the following sections: research methodology and data analysis, findings of the study, and discussion of practical implications along with limitations of this research.

## 2. Materials and Methods

The design process of the paper-based packages was intended to be in collaboration with consumers over a series of qualitative participatory focus group workshops. To achieve this, the study was divided into two stages, with Stage 1 aimed at understanding consumer expectations from sustainable paper-based packages in general and Stage 2 involved evaluation of the prototype packages designed based on findings and information obtained from Stage 1.

### 2.1. Procedure

Focus groups took place in a discussion room where participants were comfortably seated around a table so that they could see each other to allow for good interactions and discussions. Following best practices for conducting focus groups [30], each focus group session was made up of 6–8 participants, equally distributed in terms of age with two-thirds of the group being female due to the higher ratio of females:males that took part in the study. At the beginning of each session, the moderator gave an overview and stated the purpose of the study and what the role of the moderator would be. Participants were encouraged to share their opinions and were assured that there were no right or wrong answers to the questions being discussed. A pre-approved semi-structured focus group guide was used to direct the conversation. The focus group sessions lasted for approximately 2 h and were facilitated by two researchers: one moderating the session and the other taking notes. All sessions were audio- and video-recorded and transcribed verbatim for further analysis.

#### 2.1.1. Stage 1

**Figure 1:**
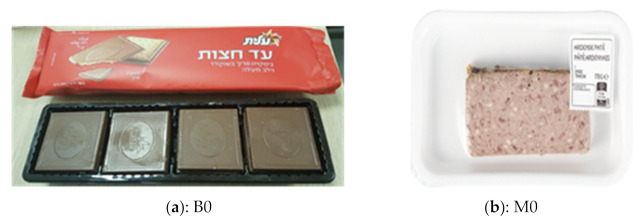
Examples of currently available packages discussed in Stage 1: (**a**) B0: Biscuit package; (**b**) M0: meat package.

**Figure 2:**
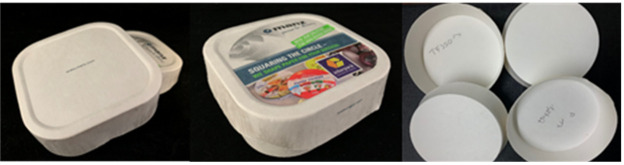
Examples of new paper-based packaging materials discussed in Stage 1.

Nine focus groups were conducted with a total of 60 participants. To get participants acquainted and comfortable with each other, foster interactions, and get them thinking about the topic to be discussed, participants were asked to introduce themselves and mention what they normally recycle. The discussions began by asking the group about their opinions of current food packaging materials available on the market. This was followed by questions around expectations and possible downsides of sustainable packaging materials. Participants were then asked to discuss considerations when buying a product. Two currently available packages (one biscuit and one meat package: Figure 1) were presented to the participants. They were asked to open, manipulate, and discuss the advantages and disadvantages of the packages. Next, participants were presented with samples of the proposed sustainable paper-based packaging material (Figure 2) and asked to give their opinions on the characteristics of the materials. Finally, participants were asked for their willingness to buy or pay more for sustainable packaging.

#### 2.1.2. Stage 2

A total of 56 participants from the first stage returned for the second stage of the study with a total of eight focus group sessions conducted. In this stage, participants were required to evaluate the paper-based packages partially designed based on their suggestions from Stage 1. The new paper-based prototype packages were presented one at a time to participants who were asked to discuss their opinions about them in terms of the design, material, etc. Participants were then asked to discuss if the packages met their expectations of a sustainable packaging material and the benefits and negatives compared to the current packages on the market. Next, they were asked to assess the ease of separation of the packaging film/barrier from the sustainable parts of the packaging. Finally, they were asked about their purchase intent of the products and how the percentage of sustainable material present in the package will influence their purchasing decision. In total, five new paper-based prototypes were developed and discussed during the session: two for the biscuits and three for the meat products (Table 1). In a life cycle assessment (LCA) performed on the paper-based trays with polyethylene terephthalate (PET) coating, the results showed that the paperboard tray has the smallest climate change impact compared to plastic crystalline polyethylene terephthalate (CPET) trays and recycled plastic recycled polyethylene terephthalate (rPET) trays. For the meat packages, a life cycle analysis screening was performed and showed that if the new-paper based packaging is recycled, while the expanded polystyrene (EPS) (M0) tray is not, the paper-based tray has the lower environmental impact (considering the paper tray is recycled ten times).

### 2.2. Participants

Participants for the study were recruited from across Berkshire, UK. Recruitment emails were sent using the University of Reading general circulation list, and the volunteer databases of the Sensory Science Centre and Nutrition Unit of the Department of Food and Nutritional Sciences, University of Reading, UK. Advertisement posters were placed on various social media platforms, local shops around Reading, UK, and on notice boards within the University of Reading, UK. Interested participants were required to complete an eligibility screener. To be eligible for the study, participants had to be: above 18 years old; not allergic or intolerant to wheat, gluten, and/or dairy; interested in food packaging; available to take part in both stages of the study. The study was conducted between April and November 2019 and approved by the School of Chemistry, Food, and Pharmacy Research Ethics committee, University of Reading, UK (study number: 11/19). Informed consent was obtained from all participants prior to the focus group sessions.

Demographic characteristics of the participants who took part in the study are presented in Table 2. A total of 60 participants took part in the study in Stage 1 with 56 returning for Stage 2. The majority of the participants were female (66.7% in Stage 1 and 71.4% in Stage 2) with the mean ages of 47 and 47.6 in Stages 1 and 2, respectively. The median age of participants was 49 in both stages with an age range of 19–71 years old. More than 60% of the participants were White British and less than 4% of Black/Caribbean/Mixed ethnicity. Almost all participants (95%) who took part in the study considered themselves environmentally conscious.

### 2.3. Data Analysis

The transcribed data and notes taken during the sessions were analyzed using content analysis. The procedure followed was similar to that used by [31]. Two researchers extracted recurring themes from the transcripts of all focus groups individually, with the summary of key findings obtained by comparing the results of each researcher. For a result to be included, it had to have been mentioned in at least four out of nine (Stage 1) or eight (Stage 2) of the sessions [31,33].

## 3. Results

The results of the focus group discussions are presented by summarizing common themes that emerged from the focus group sessions, although the participants discussed each package individually. Some comments from the discussions are included to show how participants reflected on some of the themes.

### 3.1. Stage 1

#### 3.1.1. Opinions on Available Packages Currently in the Market

The main themes highlighted by the participants in all sessions were the amount and type of packaging used to package foods. Participants stated that there was too much packaging and over-packaging of foods, most of which is unnecessary, with one participant saying, “you don’t have to have individual wrappings for everything” and another, “why have a wrapping around a coconut?”. The second point mentioned was that there was too much plastic (especially single-use plastics) packaging and black trays used to package products: “why use plastic except [when] absolutely necessary?”; “the amount of plastic being used is shocking and too much”. Some participants stated that the reason being given for too much packaging was to protect the food for consumers which is what consumers want because they are wary of contamination. Other participants argued that consumers are constantly being told that but were wondering if it is what consumers actually want or what supermarkets need: “are shops just trying to pawn the waste to consumers to increase profits?”; “the packaging is to help the shops, not the world or consumer”. Confusion on how to handle packages with more education needed was another theme highlighted across the focus group sessions: “most consumers do not understand how recycling works; do packages need to be washed before disposing them in the recycling bin?”; “clearer directions from manufacturers on how to dispose packaging is necessary”; “more universal methods of disposing packages are necessary”. Some participants felt that glass was more sustainable than plastic packaging, but others argued that the production process of glass actually makes it less sustainable which showed that consumers were confused about sustainability in terms of food packaging: “glass is not necessarily more sustainable because the cost of production of recycling glass is 80% more than using fresh products”. Participants called for full transparency of the packaging process; “we don’t have the full story”; “consumers need information on things like the carbon footprint of packaging materials”.

Overall, participants agreed that a cultural change is needed; consumers need to be more flexible with their requests on how foods are packaged; manufacturers need to change consumer attitudes and perspectives; and governments need to introduce laws which will help reduce the amount of packaging being used and give consumers no choice but to adapt. The ban on free plastic bags introduced by the UK government some years ago was highlighted as an example of how the government can help change consumer attitudes, with participants stating that more people now take their reusable bags (bags for life) with them when going to shop which has led to a sharp decline in the number of plastic bags being used. In summary, participants agreed that the “3Rs”—Reduce, Reuse and Recycle—need to be the mantra to make food packages more sustainable and environmentally friendly.

#### 3.1.2. Considerations When Buying a Product

Price was the main driving force considered by participants when buying a product and was closely followed by the product quality, with comments such as: “the first thing I think of is whether I am getting value for money”; “for me if I am buying anything, the quality of the product is at the forefront and then I consider whether I can afford it”. For most of the participants, how the product is packaged was the last thing considered during purchasing. Most people stated that they only considered that when they got home. When asked if they considered sustainability of the packaging material when making a purchase, very few consumers stated that it was on their list of considerations, with most saying that they only considered the packaging sustainability after the purchase and that it was not a driving force at the point of purchase. Other factors that influence purchase intent mentioned by participants included personal choice, habit, how much time they had to shop, and what or if alternatives were available: “if I had a choice, I will go for something in a glass instead of plastic because I feel glass is more sustainable, but sometimes you don’t have a choice”; “I sometimes try to find products in more sustainable packaging, but sometimes they are not available and because I need it urgently, I end up buying anything I see”; “it depends on how much time I have got; if I had enough time, I would look around for products packaged in a sustainable way but if I didn’t, I would just shove things into my basket without thinking of how they are packaged”. Others said, “it depends on the cost; if loose fruits were slightly more expensive than fruits packaged in a plastic bag but within my budget, I would buy the loose fruits, but if they were over my budget, I would buy the packaged fruit”; “when I go for my weekly shopping, I generally go for brands I am used to within my price range without considering the packaging”. Overall, most of the participants agreed that price and convenience trump environmental friendliness when making a purchasing decision.

#### 3.1.3. Expectations from a Sustainable Packaging

When asked to discuss expectations from a sustainable package, the main themes mentioned by participants were functionality in terms of maintaining product quality (e.g., freshness) and shelf life: “it should do its work of keeping the product safe and maintaining its quality”; durability: “It should be strong, stress-resistant, and able to keep the product intact without splitting or breaking until I get to my destination”; aesthetic value: “the design should be very attractive and stand out from other less sustainable packages”; must be recyclable or biodegradable: “a sustainable package should be easy to recycle and would be better if it was 100% recyclable”; “there is no point in me buying an attractive package if it is not recyclable”; minimal amount of packaging should be used: “do not over package products; use just enough packaging required to maintain product quality and safety”. A key point mentioned was that packages need to be clearly labeled for sustainability; “the sustainability message needs to be clear so consumers can easily see that the package is more sustainable that other packages”. Other points mentioned were that packages should be resealable (though this is product-dependent) and reusable, and that they should meet the standard requirements for the product that the packaging is being used for (e.g., oxygen and moisture barriers) and transparent where possible: “if I am buying a fresh product like meat or vegetable, I would like to be able to see what I am buying so I am sure it hasn’t gone bad”. The key characteristics outlined for a sustainable package were functionality, clear information, aesthetic value, and product shelf life. In summary, consumers expect a sustainable package to do everything a standard package would do and not be harmful to the environment (environmentally friendly) at the same time. Participants, however, agreed that this was a lot to ask, and there were some limitations in the ability of some sustainable packaging such as paper to keep foods fresh for a long period.

#### 3.1.4. Opinions on Currently Available Biscuit and Meat Packages Discussed in the Study

Participants were presented with a biscuit and a meat package currently available on the market (Figure 1) and asked to express their opinions of the packages. Results from the discussions were grouped into themes and presented based on those themes rather than on individual packages. Key themes that emerged were packaging material, design, size, functionality, and labelling. Participant responses were both positive and negative.

In terms of packaging material, participants commented on the flimsy nature of the outer wrapper of the biscuit package and the fact that it was made from foil-like material which was considered a negative with comments such as: “the wrapper rips up easily” and “oh you’ve got foil inside”. The inner packaging of the biscuit, a black plastic tray, was considered in a negative light and was said to be pretty standard. Most participants disliked the feel of the polystyrene packaging of the meat including the single-use plastic lid; “this packaging is not recyclable”. Both the biscuit and meat packages were considered harmful to the environment as they are not biodegradable, neither can they be reused or recycled. There were several suggestions on how the packages could made more environmentally friendly, with comments such as: “instead of the black plastic tray, the biscuits could be in a cardboard box”; “polystyrene! Can’t it be cardboard?”; “why not use paper packaging and have a window on the lid so the product is visible to consumers?”.

When it came to the design of the packages, participants had varying opinions on the biscuit package. While some loved the red color, found it attractive, and said it made it look expensive, others said the red color made it look cheap and unappealing and was designed to deceive the consumer; “design looks dull”; “red color of the package is designed to make us think it is a special product; if a different color was used, it won’t be as appealing”. Most participants loved the fact that the image of the product was on the packaging and that the product was not visible, stating that the images were a true reflection of the product inside. This was, however, disputed by others who felt the image was not a true reflection of the product. A small subset of participants loved the meat package and felt it gave the product a positive outlook, with comments such as: “gives the product a sense of freshness”; “looks like a packaging used in a Deli”; “looks like a product from a local butcher”; “love the transparent lid”. Most participants, however, did not like the design of the meat package and found it unappealing and unattractive, with several comments such as: “looks very boring and dodgy”; “looks cheap and nasty”; “don’t like the white color; white puts me off”; “I won’t buy this if I had an option”.

When discussing the size of the packages, participants found the size of the biscuit package generally acceptable when compared to the number of biscuits in the package and did not have much to say about it, with a few participants commenting that the packaging could be reduced a little if the biscuit was packed in a different way: “stacking the biscuits side by side like you have in some biscuits like digestive may reduce the amount of packaging used”; “instead of a separate outer foil and inner black tray, using a paper tray with a well-sealed top would have been better and reduced the amount of packaging”. On the other hand, the meat was said to have been over-packed, with comments such as “too much packaging”; “there is too much empty space in the package”; and “the package is too big for the amount of product inside” mentioned by participants.

Another theme highlighted was “package function vs. products inside”. Participants stated that the biscuit package was not very functional and did not perform the function of retaining the quality of the product: “the package is not protecting the biscuits; there are too many broken biscuits in my pack”; “package is too loose”. Participants had little or nothing to say on the functionality of the meat package but had a lot to say about the labelling, with many comments related to the size and descriptions on the label: “label occupies too much space covering the product and making it not visible to the consumer”; “disposal information not visible enough”; “label should be more visible”; “different signs on the label is very confusing and unclear”. Similar comments on the clarity of the label were made about the biscuit packaging. Participants found labelling instructions both very confusing and difficult to understand. Overall, participants preferred the biscuit package over the meat package mainly because they found the design of the biscuit package more appealing but felt both packages were not environmentally friendly and were “over-packed/over-wrapped”, and they felt that the volume of the meat package could be reduced by up to 40%.

#### 3.1.5. Opinions on Proposed Paper-Based Packaging Materials

The key themes that came out of the conversations around opinions on the proposed paper-based packages (Figure 2) were appearance, material characteristics and feel, functionality, ethical qualities, and emotional draw. The appearance of the packaging material was generally described as “looks natural”, “biodegradable” and “recyclable” which are all positive comments and characteristics expected from a sustainable packaging material. Other characteristics mentioned included: “shiny outer coating”, “looks flimsy and cheap”, “doesn’t look sturdy enough for transporting?”, “looks boring and unappealing”. Some participants worried that on the surface, the materials did not look strong enough to withstand stress: “is it strong? If you got a leak would it break?”.

On touching and manipulating the material, participants described the packages as “stretchy and flexible”, “a lot stronger than it looks”, “strong paper: not very easy to tear” and “leak proof”. Functionality was discussed in terms of the protection and preservation that the material will offer to the product packaged in it. The materials were described as “durable”, “will retain its shape with moisture”, “can be used to package both the biscuit and the meat as well as many other food products”, and “the shiny barrier or coating will cope with greasy products”.

Ethical issues mentioned were centered around the sustainability value of the products. Though participants generally agreed that the packages had environmentally friendly characteristics and commented that the packages “could be marketed as eco-friendly versions of similar products”, there were concerns around it being a single-use package with comments such as: “it is not reusable” and “it’s a one-off use package”. There were discussions around the amount of the packaging that would be recycled, with most consumers happy to separate the non-recyclable barrier from recyclable materials and satisfied if more than 50% of the package was recyclable, while others stated that “it gets confusing if not completely recyclable”. In addition, participants were worried that though the package was recyclable, it can still end up in the landfill if it is contaminated by the product inside, and they wondered at what point it gets past the stage of recycling due to contamination. Another concern about the packages was what the cost of production was, compared to current packages, as that could affect the sustainability characteristic of the package in the long run, especially as it is not reusable. The final theme discussed was around the emotional response the packages drew from consumers, with most having a positive emotional pull: “makes me feel better that part if not all of the package is recyclable”. This may have a positive impact on consumer attitudes towards sustainability.

Finally, given that most sustainable packages generally cost more than their non-sustainable counterparts, consumers were asked if they would be willing to pay more for the packages made from the sustainable paper-based material presented. While consumers welcomed the idea of replacing the current packages with the new packages, most of them were unwilling to pay more for the product saying that they expected the companies to bear the cost and could not understand why they should be charged more for doing what is right and helping the environment: “doesn’t make sense that we have to pay to be green—so consumers shouldn’t have to pay more for it”; “the increased price needs to be justified”; “companies should take it as their social responsibility”. Very few participants across the focus groups were happy to pay a maximum of 10% more for the sustainable packages but suggested that “companies must ‘sell’ it to the consumer—give incentives” and governments should make legislations forcing companies to use more sustainable packages and could introduce taxes/fines if other non-sustainable materials are used. Participants would like to see more government initiatives and incentives to reduce the use of less sustainable packaging materials: “make plastics less lucrative”.

In conclusion, consumers felt that everyone (government, manufacturers, and consumers) had a part to play if the change to sustainable packaging is to be successful.

### 3.2. Stage 2

Following evaluation of the paper-based package prototypes (Table 1) and comparing them to the old existing packages, the following themes emerged: appearance, material characteristics, design and size, functionality, target population/market, and price/purchase intent.

#### 3.2.1. Packaging Material Characteristics

Appearance, strength, and feel were the main packaging material characteristics discussed. In terms of appearance, the B2 prototype package was described as more appealing and preferred than the B1 package with comments such as “quite attractive—catches the eye” and “looks classy, like a quality product”. On the other hand, statements such as “looks cheap and unappealing” were used to describe package B1. Comparing the current biscuit package (B0) to the prototypes, participants found B0 more attractive than both B1 and B2. When discussing the appearance of the meat packages, prototypes M2 and M3 were more preferred than M1, with M1 described as looking “very amateurish”, “cheap and unattractive” and “shocking!” while M2 and M3 were described as “looking very basic in a good way”. Similar to the biscuit packages, participants preferred the appearance of the existing meat package (M0) with comments such as “it looks neater than the others”. One of the positive comments for the prototype meat packages, however, was that they looked more natural and environmentally friendly than M0.

Discussions around the strength of the packages revealed that participants found the B2 package to be “more sturdy” than B1, which was described as “very flimsy”. B2 was considered to be “more rigid and stronger” than B0. The tray strength of meat prototype package M3 was said to be the “most rigid” of all the three prototype packages with M1 and M2 described as “very flimsy” and “less sturdy” than M3, respectively. The lid strength of the three prototypes were also discussed, with participants mentioning that the lids of M1 and M3 were “stronger” and “won’t tear easily” when compared to the M3 lid, which they felt “may be easy to tear compared to the other ones”. “Looks easily breakable” and “not as strong as the paper packages” were some of the ways the M0 package was described by participants.

The final characteristic mentioned was the feel of the packages. In general, participants loved the cardboard feel of all biscuit and meat paper-based prototypes. However, the “bumpy” feel of package B2 was preferred to the smooth feel of B1 with participants stating that the “bumpy” feel of B2 gave it a “better grip” and made it easier to hold than B1. One participant described the feel of B1 as “feels cheap—don’t like it”. Statements used to describe the meat paper-based packages included: “feels natural”, “has a homemade feel, like something from the butchers”.

#### 3.2.2. Design and Size

All the biscuit and meat prototype packages were considered too big for the amount of the product they contained. While in the case of the biscuit packages, participants found the size of B0 great and just right for the amount of biscuits it contained, they said that the M0 package was too big for the portion of meat inside. Comments for the paper-based packages include “definitely a waste of space”; “why use so much packaging?”; “the fact that it is supposed to be a more sustainable package doesn’t mean it should be this big; “what a waste!”. On another note, participants felt that the shape of biscuit packages B1 and B2 needed to be modified, as the shape limited the number of biscuits that the packages could accommodate, referring to it as “not deep enough”. Participants felt that the packages were too big, with comments such as: “packaging probably cost twice the price of the biscuits”. On the other hand, while participants loved the shape of the M3 package and described M2 as “looks like a proper tray—with less packaging”, participants found the shape of the M1 package to be “too big”, “funny”, and “not well-defined”. The light weight of the paper-based prototypes was loved, with participants saying: “it is very light so will be easy to carry”.

In terms of the design, the white and red color contrast of packages B1 and B2 was loved and preferred when compared to the “all red” color of B0. In addition, participants preferred the foldable pack design of B1 and B2 to the flat design of B0, though some participants found the double pack design very confusing and felt it would be better to separate the two packs. However, the “bumpy” design of B2 was favored to the smooth design of B1. When discussing the meat packages, participants found all three prototypes, M1, M2, and M3 too plain-looking. The lid of the meat packages was discussed, with participants disliking the non-transparent paper lid of M3 because it made it impossible to see the contents of the package. Suggestions mentioned included: “put a window for product visibility”, but some participants disagreed, saying: “I need to see everything to know how the product inside looks like; a window doesn’t work for me”. Finally, participants were not impressed with the shiny barrier in the paper-based prototypes and found it off-putting, as they felt it made the packages less sustainable and more difficult to recycle with comments such as: “outer package says sustainable but inside says a different thing” and “can’t tell if it is paper or plastic”.

#### 3.2.3. Functionality

All paper-based prototype packages were said to be very difficult to open compared to B0 and M0. It was suggested that “a side flap and indicator for opening” be added, just as was present in M0 that needed to be included in the design to guide consumers on where to open the packages. However, for the biscuit packages, participants found B2 a bit easier to open than B1, which they attributed to the “bumpy” nature of the tray which made it firmer to hold. The difficulty in opening the packages was seen as a positive by the participants in some way, as they felt it meant the packages were tightly sealed, improving their preservation characteristics and making them more stress-resistant. B1 and B2 packages were said to offer more protection to the biscuits than B0 due to their rigidity and foldable design, with participants saying the B2 “bumpy” design offered more protection than the smooth B1. On the other hand, participants found it difficult to split both packages, with most splits resulting in broken biscuits and opened seals, which participants found unacceptable. It was suggested that single packs would be better than duo packs and be more functional overall. Participants were nervous about contamination in M1, M2, and M3 packages, with worries that the M2 lid was touching the product which could lead to contamination, unlike in the case of M1 and M3. There were concerns over the protection of the products inside the paper-based packages if they got wet due to rain or cold storage in the case of the meat packages, with comments such as: “what happens when it gets wet or soggy?”.

Though worried about the sustainability aspect of the barrier in M1, M2, and M3 packages, participants found it very functional in keeping the product safe. Participants found separating the barrier of the paper-based packages from the paper material difficult to varying degrees. For the biscuit packages, B2 was easier to separate than B1 while for the meat packages, M2 was the most difficult to separate. However, participants made it clear that they were unwilling to be saddled with the responsibility of separating the barrier before disposing the package. Some of the reasons given include: “it is a hassle”; “trying to separate the barrier in the meat package can lead to contamination”; and “if I am eating the biscuit on the go, you cannot expect me to separate the barrier”. Participants felt that the design and shape of the new prototypes were not very functional for the products, as they led to too much packaging with little content inside. They suggested that the shape of the biscuit packages should be changed to something such as a rectangle, which will reduce the amount of packaging used while increasing the number of biscuits inside. It was suggested that the black plastic tray design of B0 be retained, with the plastic replaced by a paper-based tray. A major functionality missing from the paper-based prototype packages according to participants was the inability to reseal the packages after opening, with many saying that the lid should be made resealable for storage purposes.

#### 3.2.4. Target Population/Market

Target population/market was one of the themes to emerge from the biscuit packaging discussions. While participants felt that the target market/population of the B0 package was very clear, they found the double pack of B1 and B2 to be very confusing, and the target market/population not clearly defined. It was obvious that B0 was targeted towards “family or party use”, but B1 and B2 were described as having no clear target, with questions and statements such as: “is it an on-the-go product?”; “package and content is too much to be an on-the-go snack”; “is it designed for one time consumption?”; “is this aimed at younger people?”; “I cannot imagine it as a snack pack, looks more like a lunch box”; “doesn’t stand out, no clear message or target”. These questions and comments clearly show the confusion of the participants. In terms of where the B1 and B2 products could be sold, airports, cinemas, street corner shops, and canteens were the suggested possible places, though the location would be dependent on the target market.

#### 3.2.5. Price/Purchase Intent

Price/purchase intent was a key theme highlighted during the discussions. While the B0 package was considered better value for money, B1 and B2 were not, with many participants saying they would probably buy them once but would not buy them again. Participants said they were generally not tempted to buy the biscuits in the new paper-based packaging but recommended the duo pack be separated into two packs and sold as single packs to improve the purchasing value with comments such as: “better to separate the two packs, think you will sell more” expressed by several participants. The M2 package was considered good value for money, but M1 was thought to be unacceptable to be introduced into the market. Participants were more open to buying the M2 and M3 packages with preference for the M2 because of the transparent lid but unwilling to buy M1 package.

With regards to purchase intent, similar to Stage 1, participants were generally not willing to pay more to be sustainable, but some were happy to pay “5 to 10%” more for the new sustainable paper-based packages, mostly because of their dislike of the polystyrene in the M0 package and black plastic tray of the biscuit package.

## 4. Discussion

The aim of this study was to understand consumers’ expectations and opinions of sustainable paper-based packaging materials and to evaluate and assess the characteristics and suitability of the developed paper-based prototype packages. The findings from this study contribute to existing knowledge on consumer opinions and reactions to sustainable packaging materials [2,6,7,8,9,12,15,30,31,34,35,36]. While past studies focus mainly on surveys, interviews, and general conversations around consumer opinions and attitudes to available sustainable packages, this study goes further by involving consumers in the design process of paper-based packages not currently available on the market, with consumers having the opportunity to interact physically with the packages, which is missing from some studies [2,7].

One of the main points highlighted for both the old and prototype biscuit and meat packages assessed in this study was the use of excessive packaging or over-packaging of the products which participants found off-putting. Previous studies carried out in several countries including the UK showed that consumers have a negative reaction to the over-packaging of foods [7,14,37,38,39]. Though the prototype packages in this study were made from sustainable paper-based materials, participants felt the oversized nature of the packages was a form of wastage and was considered bad for the environment, suggesting that the amount of packaging used should always be commensurate to the product they contain. A study investigating consumer perception of the environmental benefit of several ecological consumption patterns found that consumers believed avoiding unnecessary packaging had a strong positive impact on the environment [38]. In another study conducted by Lea and Worsley [40] examining Australians’ food-related environmental beliefs, minimal use of packaging by food manufacturers was said to be the most important way to help save the environment. Hanssen et al. [39] investigated the environmental profile of ready-to-eat meals and found that over 50% of the participants thought that the manufacturers used too much packaging. On the contrary, in another study, when asked what made a package environmentally friendly, consumers did not consider the amount of packaging as an important factor [41]. The varying positions suggest differences in consumer perception and opinions of what environmentally friendly means. As manufacturers consider moving to more sustainable packaging options, size should be an important aspect to bear in mind, as consumers consider over-sized packaging a negative characteristic of a sustainable package.

Too much plastic packaging was mentioned as a major problem in today’s food packaging, with participants discussing the negative impact of these plastics on the environment. On the other hand, participants found the paper-based prototypes as a more sustainable packaging solution to the plastic and polystyrene packages currently used for the biscuit and meat products assessed in the study. The result of this study corresponds with the findings of previous research where paper and plastic were ranked by consumers as the most and least environmentally friendly materials, respectively, when comparing plastic, paper, glass, and metal [7,9]. Consumers who took part in a study in Sweden highlighted the negative environmental impact of plastic packaging, with paper reported to be more environmentally friendly [8].

Consumers are, however, still unclear as to what the most sustainable packaging is, with their judgements being mostly subjective and based on their personal perception rather than the sustainability characteristics of the product. Discussions around sustainability in the current study showed that while some participants considered paper-based packaging to be the most sustainable packaging, others felt glass was more sustainable in the ease of recycling. This was, however, disputed by other participants who stated that the cost of recycling glass made it less sustainable and environmentally friendly than assumed. These conversations reflect the limited knowledge that consumers have on what a sustainable product is and the confusion they face when determining what a sustainable product is. Participants agreed that consumers need to be better informed and educated on the production process of packaging to help them make informed decisions. van Dam [10] and Allegra et al. [42] reported that consumers rated paper-based packaging as the most environmentally friendly material. A survey of Swedish consumers revealed that consumers based their judgement of the environmental impact of food packaging on their perception but were also aware of the flaws in their judgement [7]. These results show that there is a need for better guidance to ensure that the noble intentions of consumers to be sustainable are not unknowingly thwarted by their decisions. In general, participants defined a sustainable product based on 3Rs—Reduce, Reuse, and Recycle—which are important processes within the Circular Economy [3]. In previous studies conducted in USA, UK, Germany and China [41], Denmark [43], and Sweden [7], consumers defined a package as environmentally friendly if it was recyclable and reusable, and used the minimal amount of packaging material.

Poor communication of disposal labels was another theme to come out of the focus group discussions. Participants stated that they found disposal information and communications on packages difficult to understand, which meant they ended up not disposing the packages in the right manner in some cases, and most people found this very frustrating. Results from the study highlight the challenges that consumers face as a result of poor disposal information by the manufacturers, which may lead to the benefits of the packages being lost on consumers. A study conducted by [44] on the consumer “attitude-behavioral” intention gap in relation to sustainability found that the positive environmental impact of packages is generally poorly communicated to consumers, impacting their ability to make informed decisions. The authors further underscored the importance of communication in increasing consumer awareness and knowledge of environmental aspects of a product and their influence on the consumer purchase decision. The consumer “attitude-behavioral” intention gap was further reinforced by [11] who acknowledged that consumers’ sustainable intentions to act in a sustainable way, while honorable, do not normally translate to their actual behavior. Fernqvist et al. [8] stated that poor communication of the added benefit of a product may influence consumer expectations and future purchase intent negatively.

Similar to previous studies [15,28,30,34,44], price and product quality were found to be the main driving force of consumer purchase intent. In the current study, participants stated that packaging was not on their list of considerations when purchasing a product despite 95% of them saying they considered themselves environmentally conscious individuals and would like to see more sustainable packages on the market. Participants said that they are creatures of habit and would normally stick to familiar brands and only think of the package after purchase or when they got to their destination. Participants further stated that convenience and price trump everything else. More than 80% of consumers cited the environmental status of a food packaging as one of the main factors that influences their selection of a food product but the extent to which this factor influences their decision is unclear. According to Bech-Larsen [34], while consumers are concerned about the effects of packaging on the environment, that concern seldom influences their purchasing decision because there are more important factors considered; consumers are not good at distinguishing between packaging; and their purchasing process is habitualized. Several studies showed that sustainability comes secondary to other factors such as price, convenience, product quality, and shelf life, and thus is not a major driver of purchase [41,45].

Participants expect sustainable packages to have all the functionality of a package and be sustainable. Developing a package from a sustainable material such as paper, particularly for sensitive foods, might prove to be a challenging undertaking for the food industry, as it may be difficult to achieve a sustainable package that provides the required functionalities while maintaining the quality characteristics of the product inside. Participants were not wowed by the design and functionality of the paper-based packages studied. The main functions of a packaging identified by Lindh [46] were protection, communication, and facilitation of handling (which includes easy-to-open status, re-sealability, size, functional weight, shape, easy-to-grip status, etc.), and participants in this study felt the paper-based packaging did not meet most of these criteria. While they found the biscuit prototype packages innovative and different, the packaging did not perform the basic function of protecting the biscuits, with several broken pieces found inside the packages. In general, they felt the design of the packages were not eye-catching or attractive enough and stated that environmentally friendly packaging needs to stand out from other packaging on the shelf if it is to attract consumers. For the meat packages, the ability to see and judge the quality of the product inside was of particular importance to participants who preferred the M2 over the M3 package because even though they had exactly the same design, unlike M3, the M2 package had a transparent lid. Participants, however, stated that the requirement of a transparent lid is mainly applicable for fresh products (e.g., meat, fish, etc.) and does not apply to dry foods such as biscuits. A growing trend in the food industry is a shift away from just showing product images on the package to using transparent packaging materials, which allow consumers to see exactly what they are buying [47]. Previous studies showed that transparent packaging increases expected freshness, expected quality, and purchase intent in various food categories [48,49]. This suggests that transparent packaging has an effect on consumer behavior [47]. Participants also found the color of the meat packaging too plain and dull and were not tempted to buy these products. In addition to text and pictures, color has been shown to affect consumers’ preference for environmentally friendly products [8]. Magnier and Schoormans [2] in their study investigating consumer reactions to sustainable packaging across two countries and products found that attractiveness and visual appearance were important factors to consider when designing environmentally friendly packaging, as this was strongly correlated with increased preference and purchase intent.

Though previous studies [7,27,41] showed that environmentally conscious consumers are often willing to pay more for environmentally friendly products, most participants in this study, though they considered themselves environmentally conscious, were unwilling to pay more for the sustainable paper-based packaging. A few people were, however, willing to pay 10–15 pence more for the sustainable paper-based packaging but stated that the packaging did not currently meet their expectations in terms of design and functionality and would have to do so if they were to pay more. 

The findings of our study corresponds with the study of Ertz et al. [13], where consumers were unwilling to pay more for more sustainable packaging, and Barber [13,50], where only 28% of consumers were willing to pay more for environmentally friendly “green” wine packaging. Krystallis and Chryssohoidis [51] found that consumers are unwilling to pay more for packaging that they do not believe meets their standards. While most studies show that consumers are willing to pay more for sustainable packaging, the amount they are willing to pay varies between studies and is difficult to measure because of the difference in packaging products studied and how the cost is presented.

This study is not without its limitations. Firstly, not all of the information obtained during Stage 1 was considered in the development of the paper-based prototypes as a result of the technology used and the geometries that could be realized with the paper-based material. The prototypes developed did not have any labelling information, so this aspect was not considered in Stage 2 of the study. On another note, more than 65% of the participants that took part in the study were female which may have biased the results of the study. Previous studies showed that females have a more positive attitude towards the environment and care more about sustainable food packaging than males [23,28].

## 5. Conclusions

This study provides further understanding of consumer responses and opinions to sustainable paper-based packaging. While the results of this study highlight key consumer opinions of a sustainable paper-based package within the UK population, we recognize that findings may differ with a larger sample size or different demographic within the UK or in other parts of the world due to cultural and regional differences regarding sustainability perception of consumers. Focus groups have been reported as a good way to gain insights into consumer opinions regarding issues which can then be analyzed using a more quantitative methodology in the future [8]. The result of this study shows that participants who took part in the study are (i) aware of the environmental impacts of food packages; (ii) concerned about the negative impact of the unsustainable packages on the environment, and (iii) desire a change in the type and amount of materials used in food packaging. This study further confirms that price and quality remain key driving forces for consumers’ purchase intent. Participants did not like the paper-based packages evaluated in this study but found the biscuit design interesting and innovative. Overall, the paper-based packages did not meet participants’ expectations, but they all agreed that the design was headed in the right direction. To validate the results of this study, a quantitative study with 130 participants was conducted with results corresponding with this study [49]. In summary, the key message that emerged from the discussions was the “3Rs”—Reduce, Reuse, and Recycle —which should be the main points to consider when designing a sustainable packaging. In addition, a cultural change is needed across all stakeholders (government, manufacturers, and consumers) if success is to be achieved.

## Figures and Tables

**Table 1:** Biscuit and meat packages discussed in Stage 2.

Code	Packaging Description	Image
Biscuit packages		
B0	Preformed polymer multicavity tray, polymer flow pack (horizontal)	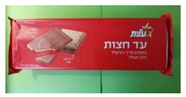
B1	Form-fill-seal paper-based tray with paper-based lidding film and smooth tray surface	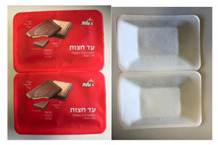
B2	Form-sill-seal paper-based tray with paper-based lidding film and embossed surface	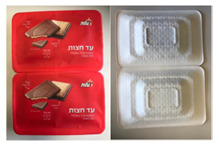
Meat packages		
M0	Preformed polymer tray with polymer lidding film with opening flap	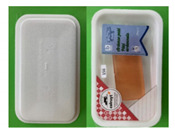
M1	Preformed paper-based tray with polymer lidding film identical to M0 with more depth and transparent polymer lidding film.	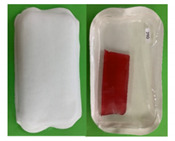
M2	Form-sill-seal paper-based tray with polymer lidding film, smooth tray, and less depth with transparent polymer lidding film.	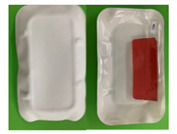
M3	Form-fill-seal paper-based tray with paper-based lidding film, embossed tray bottom and non-transparent paper-based lidding film.	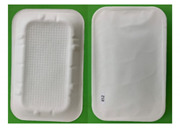

**Table 2:** Demographic characteristics of focus group participants.

	Stage 1		Stage 2	
Participant Characteristics	Number	Percentage (%)	Number	Percentage (%)
Total number of participants	60		56	
*Age (years)*				
mean	47		47.6	
median	49		49	
min	19		19	
max	71		71	
*Gender*				
male	20	33.3	16	28.6
female	40	66.7	40	71.4
*Ethnicity*				
Asian/mixed Asian	11	18.3	8	14.3
Black African/Caribbean/Mixed	2	3.3	2	3.6
White British	39	65.0	39	69.6
White other	8	13.3	7	12.5
*Environmentally conscious*				
yes	57	95.0	53	94.6
no	3	5.0	3	5.4

## Data Availability

The data presented in this study are available on request from the corresponding author.
